# The impact of exercise on spinal posture in adolescents: a systematic review

**DOI:** 10.1590/1984-0462/2025/43/2025013

**Published:** 2025-11-14

**Authors:** Ercília Oliveira-Costa, Diego Alonso-Fernández, Águeda Gutiérrez-Sánchez

**Affiliations:** a Universidade de Vigo, Pontevedra, Spain.

**Keywords:** Posture, Adolescents, Spinal curvatures, Physical exercise, Postura, Adolescentes, Curvaturas da coluna vertebral, Exercício físico

## Abstract

**Objective:**

The objective of this study was to analyze research evaluating the impact of various physical exercise programs on improving spinal posture in adolescents.

**Methods:**

A systematic review covering the last six years (2018–2024) was conducted following the Preferred Reporting Items for Systematic Reviews and Meta-Analyses (PRISMA) guidelines across nine databases. Methodological quality was assessed using the Physiotherapy Evidence Database (PEDro) scale.

**Results:**

A total of 152 studies were identified, of which ten met the selection criteria and demonstrated high methodological quality and were included in the review. These studies showed a reduction in the thoracic and lumbar kyphosis angles, lumbar lordosis, and the percentage of pain, as well as an increase in trunk mobility and inclination angle following the implementation of physical exercise programs.

**Conclusions:**

In intervention groups physical exercise has an impact on posture, leading to significant improvements in thoracic and lumbar kyphosis angles compared to control groups.

## RESUMO

**Objetivo:**

O objetivo deste estudo foi analisar pesquisas que avaliaram o impacto de diferentes programas de exercício físico na melhoria da postura da coluna vertebral em adolescentes.

**Métodos:**

Foi realizada uma revisão sistemática dos últimos seis anos (2018–2024), de acordo com as diretrizes *Preferred Reporting Items for Systematic Reviews and Meta-Analyses (PRISMA)*, em nove bases de dados. A qualidade metodológica foi avaliada por meio da escala Physiotherapy Evidence Database (PEDro).

**Resultados:**

Foram identificados 152 estudos, dos quais dez foram incluídos na revisão por cumprirem os critérios de seleção, apresentando elevada qualidade metodológica. Esses estudos demonstraram diminuição do ângulo de cifose torácica e lombar, da lordose lombar e da percentagem de dor, bem como aumento da mobilidade do tronco e do ângulo de inclinação após a implementação dos programas de exercício físico.

**Conclusões:**

Nos grupos de intervenção, o exercício físico tem impacto na postura, levando a melhorias significativas nos ângulos da cifose torácica e lombar em comparação com os grupos de controle.

## INTRODUCTION

 Public health issues, such as postural changes and back pain, affect not only adults but also children and adolescents. From childhood, bodily morphological changes arise as a consequence of postural changes,^1^ where postural balance is achieved when body alignment ensures maximum physiological and biomechanical efficiency.^2^

 There is a higher prevalence of lower back pain in increasingly younger individuals, especially during school age.^3^ The intensity of pain is generally lower until the age of ten, becoming more significant after that age, reaching pain intensity levels at 15 comparable to those in adults.^4^ Thus, starting prevention from the early school years allows the establishment of adequate movement patterns, avoiding the need to correct these patterns and inadequate postural habits later.^5^

 Previous studies show that between 22 and 65% of adolescents adopt an inadequate posture,^6, 7^ and it is essential to understand the interconnection between balance and posture in younger age groups, as well as its interaction with other musculoskeletal conditions.^8^ The adoption of improper postures for long hours, both standing and sitting, associated with the prevalence of sedentary behavior, becomes a significant factor contributing to the early development of postural deviations in young people,^9^ such as increased thoracic kyphosis.^10^ This deformity can arise due to spinal misalignment across all age groups,^11,12^ resulting in pain and postural changes and the consequent need for appropriate interventions.^13^ Likewise, spinal misalignment is associated with slower gait, lack of balance, and greater susceptibility to falls.^14^ Thirty percent of school-aged children have spinal deformities that may lead to static deformities.^7^ Such static deformities can result in severe postural disorders in adulthood if timely corrections are not made.^15,16^

 Different authors have highlighted the importance and impact of exercise on spinal posture.^14^ The practice of appropriate exercises can prevent various postural effects and spinal deformities, promoting a more uniform body function during adolescence,^15^ as continuous and systematic exercise can influence the sagittal curvature of the spine.^14^ On the other hand, low levels of hamstring flexibility and lack of abdominal and paravertebral strengthening are directly related to hyperkyphosis, while hyperlordosis is associated with shortening of the iliopsoas muscle.^17^

 In addition, mobilization in dorsal spinal extension serves as an intervention that itself improves thoracic alignment.^18^ Thus, corrective exercises demonstrate a significant impact in addressing anomalies (forward head projection, shoulders forward, kyphosis, and scapulohumeral rhythm), and stretching and strengthening exercises for the thoracic and posterior trunk muscles contribute to a decrease in the thoracic kyphosis angle.^19^

 Although several studies have examined the effects of physical exercise on spinal posture in adolescents, important limitations persist in the current literature. Many of these investigations rely on small sample sizes, lack control groups, or use heterogeneous intervention protocols and assessment tools, making it difficult to draw consistent conclusions. Furthermore, most studies are conducted in clinical contexts, with limited applicability to school settings, where postural deviations are highly prevalent. 

 Another relevant gap is the lack of studies specifically focused on the impact of exercise programs aimed at preventing or correcting postural changes during adolescence, a critical developmental period marked by musculoskeletal growth and the consolidation of postural patterns. 

 Therefore, there is a clear need to systematize the available evidence regarding the effects of physical exercise on spinal posture in healthy adolescents, especially concerning sagittal curvature parameters such as thoracic kyphosis, lumbar lordosis, and spinal mobility. 

 This systematic review aims to address this gap by identifying and critically analyzing the most recent studies on exercise-based interventions designed to improve posture among adolescents. Despite the recognized potential benefits of physical activity, the existing literature still lacks robust evidence on the application of postural corrective exercises specifically during adolescence. This review seeks to consolidate current scientific knowledge and support evidence-based strategies for postural health promotion in this population. 

## METHOD

 The analysis methods, inclusion criteria, and research strategies for this systematic review were included and registered in the International Prospective Register of Systematic Reviews (PROSPERO; code CRD42023494743), following the guidelines of the *Preferred Reporting Items for Systematic Reviews and Meta-Analyses* (PRISMA) methodology.^20^

 A systematic research was conducted in July 2024, including results from the last six years (2018–2024). The time frame from 2018 to 2024 was defined to ensure the inclusion of the most recent and methodologically robust evidence, considering the rapid evolution of research related to adolescent postural health and exercise-based interventions. This period also aligns with the publication of updated global health guidelines by the World Health Organization (WHO) and Pan American Health Organization (PAHO), which emphasize the promotion of physical activity during adolescence. Furthermore, this six-year window enables the identification of trends in the application of postural exercise programs in line with current educational and public health contexts, ensuring the relevance of the findings to contemporary practice. Terms related to the population and intervention were used, combining standardized Medical Subject Headings (MeSH) terms and free-text terms in accordance with the recommendations of the Cochrane Handbook for Systematic Reviews of Interventions.^21^ Table 1 shows the equations used in each database as well as the keywords. 

**Table 1:** Search queries in the different databases.

Database	Query
1. Web of Science Core Collection	(ALL=(Posture) OR (Body position or placement) AND (Exercise) OR (Exercise Movement Techniques) OR (Physical Activities) OR (Exercises) OR (Body Practices) OR (Physical Training) AND ALL=(Adolescent) OR (Adolescence) OR (Adolescents) OR (Young) OR (Young people) OR (Youth) OR (Teenager) OR (Teenagers) OR (Teens) AND ALL=(Spinal Curvatures)OR (Defective curvatures of the spine)OR (spinal deviations) and 2018 or 2019 or 2020 or 2021 or 2022 or 2023 or 2024 (Publication Years).
2. MEDLINE/PubMed	(Posture) OR (Body position or placement) AND (Exercise) OR (Exercise Movement Techniques) OR (Physical Activities) OR (Exercises) OR (Body Practices) OR (Physical Training) AND (Adolescent) OR (Adolescence) OR (Adolescents) OR (Young) OR (Young people) OR (Youth) OR (Teenager) OR (Teenagers) OR (Teens) AND (Spinal Curvatures) OR (Defective curvatures of the spine) OR (spinal deviations) Filters: from 2018–2024
3. LILACS (BVS)	(posture) AND (exercise) OR (physical activities) OR (exercises) OR (physical training) AND (adolescent) OR (adolescence) OR (adolescents) OR (young) OR (young people) OR (youth) OR (teenager) OR (teenagers) OR (teens) AND (spinal curvatures) OR (defective curvatures of the spine) OR (spinal deviations) AND (db: (LILACS).
4. PEDro	Exercise adolescents Posture
5. CINAHL (EBSCOhost)	(MH "Posture" OR "Body position or placement") AND ("Exercise" OR "Physical Activity" OR "Exercises" OR "Body Practices" OR "Balance Training, Physical") AND (MH "Adolescence" OR "Adolescents") AND (MH "Spinal Curvatures" OR "Defective curvatures of the spine" OR "spinal deviations").
6. Scopus	(ALL (posture) AND ALL (exercise) OR (physical AND activities) OR (exercises) OR (physical AND training) AND ALL (adolescent) OR (adolescence) OR (adolescents) OR (young) OR (young AND people) OR (youth) OR (teenager) OR (teenagers) OR (teens) AND ALL (spinal AND curvatures) OR (defective AND curvatures AND of AND the AND spine) OR (spinal AND deviations))AND PUBYEAR>2017 AND PUBYEAR<2025.
7. ERIC	(Posture) AND (Exercise) AND (Adolescent) AND (Spinal Curvatures)
8. SportDiscus (EBSCOhost)	(MH "Posture" OR "Body position or placement") AND ("Exercise" OR "Physical Activity" OR "Exercises" OR "Body Practices" OR "Balance Training, Physical") AND (MH "Adolescence" OR "Adolescents") AND (MH "Spinal Curvatures" OR "Defective curvatures of the spine" OR "spinal deviations").
9. Cochrane Library	(Posture) AND (Exercise) AND (Adolescents) AND (Spinal Curvatures).
10. ScienceDirect	(Posture) AND (Exercise) AND (Adolescents) AND (Spinal Curvatures).

MEDLINE: Medical Literature Analysis and Retrieval System Online; PubMed: United States National Library of Medicine; LILACS: Latin American and Caribbean Health Sciences Literature; PEDro: Physiotherapy Evidence Database; CINAHL: Cumulative Index to Nursing and Allied Health Literature; ERIC: Education Resources Information Center.

 To define the eligibility criteria, the Population, Intervention, Comparison, Outcomes, Study design (PICOS) strategy was used: (P) specifically focused on the adolescent population (aged ten to 19 years); (I) studies implementing exercise programs and/or postural reeducation and their effects on spinal posture; (C) including pretest and posttest in intervention and control groups; (O) assessing the impact of exercise programs on posture; (S) experimental or quasi-experimental studies published in English, Spanish, or Portuguese. 

 Inclusion criteria were publications from 2018 to July 2024 and alignment with the study topic. Only studies in which the outcomes were specifically evaluated in adolescents (aged ten to 19 years) were included, ensuring that the intervention effects reflected this target population. Studies where participants were over 19 years old, that involved therapeutic interventions, that were in languages other than those specified in the inclusion criteria, or that did not evaluate postural effects with the application of exercises were excluded. In this review, therapeutic interventions were defined as clinical approaches such as manual therapy, electrotherapy, or pharmacological treatments. These were excluded to distinguish them from structured physical exercise programs, which aim to improve posture through movement, strength, and mobility, and are typically applied in non-clinical settings such as schools, community programs, or supervised exercise sessions. 

 The suitability of the studies was independently assessed by two authors (E.O.C. and A.G.S.) and subsequently agreed upon with the help of a third author (D.A.F.). 

 The methodological quality of the included studies was assessed using the Physiotherapy Evidence Database (PEDro) scale (https://PEDro.org.au/), which has shown high reliability and validity.^22-24^ This scale comprises 11 items that examine external validity (item 1), internal validity (items 2–9) of controlled trials, and the presence of sufficient statistical information to interpret results (items 10–11). Item 1 was not included in the quality rating of the study, as it is not used for the calculation of the total score. Each satisfied item was awarded 1 point, and each unmet item received 0 points. Summing all items on the PEDro scale provides a range from 0 to 10 points (0 points represent a high risk of bias, while 10 points indicate a low risk of bias). Quality interpretation was as follows: ≤3 low quality; 4–5 moderate quality; 6–10 high quality.^25^

**To better present the results, the included studies were grouped according to the type of intervention applied and the primary outcomes assessed:**
1. Spinal curvatures (thoracic kyphosis and lumbar lordosis),
2. Spinal mobility and alignment, and
3. Pain or postural perception.

## RESULTS

**Figure 1:**
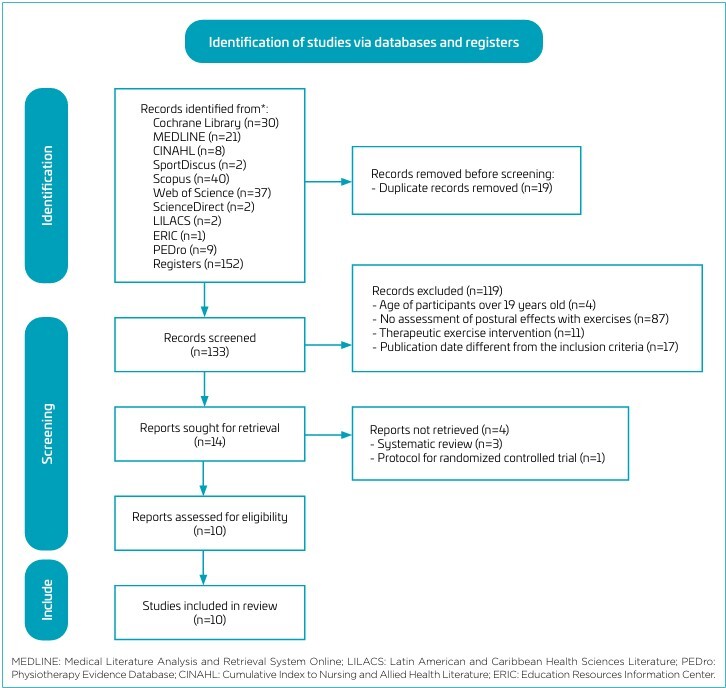
Flow diagram of the systematic review process.

 The systematic search process in the databases revealed a total of 152 studies. After removing duplicates and applying the inclusion and exclusion criteria, a total of ten full-text articles were selected. Figure 1 shows the PRISMA flow diagram detailing the study selection steps. 

 Given the nature of physical exercise interventions, blinding both participants and therapists is unlikely, as it is not feasible to blind both during the execution of exercises. Table 2 ^11,14,19,26 -32^ shows the methodological quality scores for the selected studies, with a maximum score of 7 points. All studies included group comparisons, with homogeneous groups, and provided point estimates and variability measures. Six articles were rated as high quality, and four as moderate quality, according to the criteria of Stojanović et al.^25^

**Table 2:** Results of the Methodological Quality Assessment of Included Studies — Physiotherapy Evidence Database (PEDro).

Studies	Criteria											Point	Rating
	1^*^	2	3	4	5^*^	6^*^	7	8	9	10	11		
Shadi et al.^28^	-	1	0	1	0	0	0	1	1	1	1	6	High
Firouzjah et al.^19^	-	1	0	1	0	0	0	1	1	1	1	6	High
Yang^29^	-	1	0	1	0	0	0	1	1	1	1	6	High
Elpeze and Usgu^26^	-	1	0	1	0	0	0	0	0	1	1	4	Moderate
González-Gálvez et al.^30^	-	1	0	1	0	0	1	1	1	1	1	7	High
González-Gálvez et al.^14^	-	1	0	1	0	0	0	0	1	1	1	5	Moderate
López-Ruiz and López-Miñarro^27^	-	1	1	0	0	1	0	1	0	1	1	6	High
Batistão et al.^31^	-	1	0	1	0	0	0	0	1	1	1	5	Moderate
Katarzyna et al.^32^	-	1	0	1	0	0	0	1	0	1	1	5	Moderate
Feng et al.^11^	-	1	0	1	0	1	0	1	1	1	1	7	High

Legend: 1. Eligibility criteria; 2. Random allocation; 3. Concealed allocation; 4. Group homogeneity; 5. Blinded participants; 6. Blinded therapists; 7. Blinded assessors; 8. Adequate follow-up (more than 85% of participants); 9. Intention-to-treat analysis; 10. Between-group comparisons; 11. Point estimates and variability.

* Criteria not considered for the total score (Maximum Score 10 points).

 The studies were published between 2018 and 2024 and conducted in various countries, including Brazil, Iran, Poland, Turkey, and China. Participant ages ranged from 10.5 to 16.8 years. Three studies included only male participants,^19,26 -27^ one included only females^28^ and the remaining studies involved both sexes in balanced samples. Table 3 ^11,14,19 ,26-32^ summarizes the main characteristics of the included studies, including the population profile, intervention protocols, assessment instruments, and main outcomes. 

**Table 3:** Summary of characteristics of the articles included in the systematic review.

Studies	Characteristics	Evaluation instruments	Intervention (duration and frequency)
Shadi et al.^28^	45 adolescents ♀ (average 14.06–15.00 years old); PG=15/CE=15/AT=15	Postural Evaluation: Flexicurve ruler; Hoppenfeld method	3x/60 min/week; 6 weeks of intervention
Firouzjah et al.^19^	30 adolescents ♂ (average 16.46–16.80 years old); CG=15/EG=15	Weighing scale – Postural Evaluation: Flexible ruler; Photograph; AutoCAD software; Tape measure; Scapulohumeral slip test; CKCUEST	3x/30-70 min/week; 10 weeks of intervention
Yang^29^	60 adolescents (average 15.13–15.45 years); 30♀/30♂	Measurement of body indexes – Postural Evaluation: Physical fitness assessed by pre- and post-intervention tests: sit-and-reach, 50 and 1,000 m run, lung capacity, standing long jump, and timed ascent.	2x/45 min/week; 24 weeks of intervention
Elpeze and Usgu^26^	62 adolescents ♂ (average 13.5–14.7 years old); CG=19/CCEP=21/TEP=22	Skin markers; Pedobarography – Postural Evaluation: Flexible ruler; Smartphone inclinometer	3x/40-50 min/week; 12 weeks of intervention
González-Gálvez et al.^30^	97 adolescents (average 13.48 years old); CG=48/PG=49	Relaxed standing position and sit and reach position; A-SLR and P-SLR tests; Postural Evaluation: Spinal Mouse system	2x/15 min/week; 38 weeks of intervention
González-Gálvez et al.^14^	236 adolescents (average 13.15 years old); CG=118/ EG=118; 112♀/124♂	Stadiometer – Relaxed and active standing positions; A-SLR and P-SLR tests; TT; Postural Evaluation: PAQ-A; Spinal Mouse system	2x/15 min/week; 38 weeks of intervention
López-Ruiz and López-Miñarro^27^	62 adolescents ♂ (average 16.05 years old)	PRL test and distance achieved in the sit-and-reach test; Postural Evaluation: Spinal Mouse system; Sagittal arrangement of the spine in relaxed standing and in the maximum trunk flexion position in the sit-and-reach test	2x/70 sec/week; 5 weeks of intervention
Batistão et al.^31^	171 adolescents (average 11.5 years old); CG=94/ EG=77; 115♀/56♂	Standardized demographic form; Scale; Reflective markers; Gyratory platform; Digital camera; Tripod; Postural Evaluation: Nordic Musculoskeletal Questionnaire; Questionnaire with body diagrams; Plumb line; Software (PAS/SAPO); Photogrammetric technique	2x/50 min/week; 8 weeks of intervention
Katarzyna et al.^32^	152 adolescents (average 10.5 years old); CG=76/EG Karate=76; 32♀ /120♂	Mobile stadiometer; Electronic Scale; Dermatograph; Postural Evaluation: Inclinometer	2x/60 min/week; 52 weeks of intervention
Feng et al.^11^	164 adolescents (average 13.5–14.2 years old); CG=83/EG=81	Postural Evaluation: Spinal Mouse system; Adam’s Test; Forward flexion; Matthiass test	2x/15-20 min/week; 8 weeks of intervention

Legend: PG: Pilates Group; CE: Corrective Exercises; AT: Alexander’s Technique Group; CG: Control Group; EG: Experimental Group; CKCUEST: Closed Kinetic Chain Upper Extremity Stability Test; CCEP: Corrective Compressive Exercise Program; TEP: Thoracic Exercise Program; A-SLR: Active Straight Leg Raise; P-SLR: Passive Straight Leg Raise; TT: Toe-Touch; PAQ-A: Physical Activity Questionnaire for Adolescents; PRL: Passive straight leg raising.

♂ men;

♀ women.

 Regarding spinal curvatures, several studies focused on the impact of interventions on sagittal spinal alignment. Firouzjah et al.^19^ observed significant improvements in forward head angle (Δ=-4.1^°^), forward shoulder angle (Δ=-3.0^°^) and thoracic kyphosis (Δ=-3.4 ^°^) in the experimental group (p<0.01), suggesting that the corrective exercise protocol was effective in promoting upper body postural alignment. González-Gálvez et al.^30^ reported significant adjusted between-group differences favoring the Pilates group in thoracic curvature (Δ=-5.6^°^, p=0.003) and pelvic tilt (Δ=-2.9^°^, p=0.03). The same authors also found significant intragroup reductions in thoracic curvature (Δ=-5.9^°^, p<0.001) and improvements in lumbar angle (Δ=+4.0^°^, p=0.001) in a relaxed standing position. Furthermore, significant between group differences were observed in thoracic (p=0.003) and lumbar angles (p=0.001), reinforcing the impact of Pilates on spinal alignment and postural control. Their second study^14^ reported that participants in the control group experienced a significant increase in thoracic curvature in the relaxed standing position (Δ=+5.98^°^, p=0.000), while no significant change was observed in the experimental group (Δ=+0.53 ^°^, p=0.544). Conversely, the experimental group showed improvements in lumbar curvature (Δ=+3.63^°^, p=0.000) and pelvic tilt (Δ=-2.25 ^°^, p=0.008), indicating the effectiveness of the Pilates intervention in maintaining postural alignment and preventing spinal deterioration during adolescence. 

 Shadi et al.^28^ found no significant changes in forward head (FH), rounded shoulders (RS), or kyphosis (K) in the general group (Δ=-1.9^°^, -0.6^°^, and -1.0^°^, respectively; p>0.05). In contrast, significant improvements were observed in both the corrective exercise group (FH Δ=-6.3^°^, RS Δ=-5.5 ^°^, K Δ=-6.4^°^; p<0.001) and the Alexander technique group (FH Δ=-5.9^°^, RS Δ=-4.7^°^, K Δ=-5.3^°^; p<0.01), underscoring the relevance of targeted interventions for postural correction. Katarzyna et al.^32^ observed significant structural differences between groups, with the karate group exhibiting a lower sacral inclination (Δ=-4.68^°^; p<0.0001) and greater intervertebral space between Th12 and L1 (Δ=+2.29^°^; p=0.023). These results suggest long-term karate practice may influence pelvic alignment and thoracolumbar mobility, contributing to postural adaptations. López-Ruiz and López-Miñarro^27^ reported modest changes in thoracic curvature (Δ=2.12^°^; p=0.039) and pelvic retroversion (Δ=2.11^°^; p=0.003), limited to the maximum trunk flexion position, suggesting that static posture improvements were minimal, and the effects may be posture-specific rather than generalizable to daily standing positions. 

 When addressing spinal mobility and alignment, Yang^29^ reported significant improvements in orthostatic posture, muscular strength, flexibility, coordination, and endurance (p<0.01). Among boys, increases were observed in trunk flexion (+4.9 cm), long jump distance (+26.7 cm), lung capacity (+613 ml), and pull-ups (+5.5 reps), with performance improvements in 50-meter sprint (−0.65 s) and 1,000-meter run time (-0.74 min). Among girls, gains were found in trunk flexion (+5.4 cm), long jump (+20.8 cm), lung capacity (+786.5 mL), and sit-ups (+10.6 reps), with better times in the 50-meter sprint (-1.0 s) and 1,000-meter run (-0.95 min). Feng et al.^11^ reported significant improvements in the experimental group, including a reduction in the thoracic kyphosis angle (Δ=-8.78^°^, p<0.0001), a decrease in the inclination angle (Δ=-3.31^°^, p<0.0001), and in the sacral angle (Δ=-3.27^°^, p<0.0001). Additionally, there was an increase in thoracic range of motion (Δ=+7.97^°^, p=0.003), indicating enhanced postural control and segmental mobility. Elpeze and Usgu^26^ also observed significant reductions in thoracic kyphosis in both the comprehensive corrective exercise program (CCEP: Δ=-8.93^°^, η^2^=0.304; p=0.000) and the thoracic exercise program (TEP: Δ=-4.33^°^, η^2^=0.247; p=0.001), alongside a substantial improvement in postural perception in the CCEP group (Δ=+10.48, η^2^=0.580; p=0.000), suggesting strong cognitive-motor integration effects from structured corrective interventions (Table 4)^11,14 ,19,26 -32^. 

**Table 4:** Summary of the results obtained from the articles.

Studies	Results and conclusions	Δ/Effect Size
Shadi et al.^28^	CE and AT: ↓FH, RS, k (p<0.01); PE: no significant change.	CE: FH Δ=-6.3^°^, RS Δ=-5.5^°^, K Δ=-6.4^°^ AT: FH Δ=-5.9^°^, RS Δ=-4.7^°^, K Δ=-5.3^°^ PE: FH Δ=-1.9^°^, RS Δ=-0.6^°^, K Δ=-1.0^°^
Firouzjah et al.^19^	↓FHA, FSA and k (EG; p<0.01).	FHA Δ=-4.1^°^, FSA Δ=-3.0^°^, K Δ=-3.4^°^
Yang^29^	↑Orthostatic posture, muscular strength, flexibility, coordination and endurance (p<0.01).	♂Flexion +4.9 cm, 50 m -0.65 s, 1,000 m -0.74 min, Lung +613 mL, Jump +26.7 cm, Pull ups+5.5; ♀ Flexion +5.4 cm, 50 m -1.0 s, 1,000 m -0.95 min, Lung +786.5 mL, Jump +20.8 cm, Sit ups+10.6
Elpeze and Usgu^26^	↓Angle of thoracic kyphosis (CCEP and TEP) ↑Postural perception (CCEP).	KA: CCEP Δ=-8.93^°^ (p=0.000, η^2^=0.304), TEP Δ=-4.33^°^ (p=0.001, η^2^ =0.247) PPT: CCEP Δ=+10.48 (p=0.000, η^2^ =0.580)
González-Gálvez et al.^30^	↓Thoracic angle in relaxed standing position (adjusted between groups); ↓lumbar lordosis within PG; ↑ Thoracic curvature and pelvic tilt in sit-and-reach (PG); only pelvic tilt showed significant adjusted difference between groups; ↓lumbar lordosis (PG and CG).	Thoracic angle (adjusted Δ=-5.6^°^, p=0.003); Pelvic tilt (adjusted Δ=-2.9^°^, p=0.03); Lumbar angle (Δ=+4.0^°^, p=0.001); A-SLR/P-SLR (Δ=+6.4^°^ to +15°, p<0.0001)
González-Gálvez et al.^14^	↑Thoracic kyphosis in a relaxed standing position CG; ↓Lumbar curvature and pelvic tilt in relaxed standing position EG	Thoracic kyphosis (CG): Δ=5.98^°^, p=0.000; Lumbar curvature (EG): Δ=+3.63^°^, p=0,000; Pelvic tilt (EG): −2.25^°^, p=0.008
López-Ruiz and López-Miñarro^27^	↓Thoracic curve in the posttest; ↓Pelvic tilt at posttest. There was no significant difference between the pre- and posttest in the relaxed standing position.	Thoracic curve: Δ=-2.12^°^, p=0.039, Cohen’s *d* =0.22 Pelvic tilt: Δ=+2.11^°^, p=0.003, Cohen’s *d*=0.16
Batistão et al.^31^	IG presented lower rate of worsening in shoulder posture (p=0.04); ↑Percentage of improvement for the musculoskeletal pain (p=0.04); ↓Trunk mobility (CG), ↑Trunk mobility (IG) non- significant.	Trunk mobility (CG): 1,8^º^ Trunk mobility (IG): 5,0 ^º^
Katarzyna et al.^32^	↓Sacral inclination (Karate group) ↑Th12–L1 intervertebral space (Karate group).	ALPHA 1 (sacral inclination): Karate Δ=-4.68^°^, p<0.0001 BETA Th/L (Th12–L1 space): Karate Δ=+2.29^°^, p=0.023
Feng et al.^11^	↓Angle of thoracic kyphosis CG and IG. ↓Incline angle in both groups (anterior shift); ↑Thoracic ROM (IG); ↓Sacral angle in CG and IG.	KA CG (Δ=-3.88^°^) and IG (Δ=-8.78^°^), p<0.0001; Sacral angle in IG (Δ=-3.27^°^), p<0.0001; and CG (Δ=-1.36^°^), p<0.01; Thoracic ROM (IG): Δ=+7.97^º^, p=0.003

Legend: CE: Corrective Exercise; AT: Alexander’s Technique; FH: Forward Head; RS: Rounded Shoulder; PE: Pilates Exercise; K: Kyphosis; FHA: Forward head angle; FSA: Forward shoulder angle; EG: Experimental Group; CCEP: Comprehensive Corrective Exercise Program; TEP: Thoracic Exercise Program; KA: Thoracic kyphosis angle; PPT: Postural perception training; A-SLR: Active Straight Leg Raise; P-SLR: Passive Straight Leg Raise; PG: Pilates Group; CG: Control Group; IG: Intervention Group; KG: Karate Group; ROM: Range of Motion.

↑ Significant improvement

↓ Decrease;

♂ Boys;

♀ Girls.

 Regarding pain reduction and postural perception, Batistão et al.^31^ demonstrated a greater improvement in shoulder posture (p=0.04) and pain perception (p=0.04) in the intervention group. Although trunk mobility improved by 5^°^ in the intervention group and declined by 1.8^°^ in the control group, the between-group differences were not statistically significant, suggesting possible individual variability or short-term effect. Studies that included postural education components reported perceived improvements in body awareness and self-correction strategies, reinforcing the potential value of combining physical and educational strategies in posture-focused interventions. 

 In summary, although most studies reported statistically significant improvements post-intervention, few provided measures of effect size or long-term follow-up. Therefore, while results suggest positive effects on spinal posture and related variables, the lack of standardized effect size reporting and contextual clinical impact limits broader generalizations. 

## DISCUSSION

 This systematic review aimed to evaluate the effects of exercise interventions on spinal posture in adolescents. According to the eligibility criteria, this is one of the first systematic reviews to focus exclusively on adolescents aged ten to 19 years, applying only experimental and quasi-experimental studies that evaluated the impact of structured exercise programs. The analyzed interventions included corrective exercises, Pilates, functional training, stretching routines, and school-based physical education programs. 

 The studies consistently demonstrated statistically significant improvements in spinal posture, particularly in thoracic kyphosis, lumbar lordosis, pelvic tilt, and postural mobility. These findings reinforce the importance of physical activity in this population and support the recommendations from the WHO and PAHO, which advocate at least 60 minutes of moderate to vigorous physical activity daily, including strengthening exercises three times per week.^33,34^

 However, the results must be interpreted with caution due to several methodological limitations. One of the main weaknesses is the limited sample size in many studies and the lack of stratification by sex, biological maturity, or baseline postural condition. Although some studies focused exclusively on males^19,26 ,27^ or females,^28^ most did not analyze sex-specific responses. This is relevant, as studies show that postural deviations such as kyphosis and lordosis differ by sex, with greater kyphotic angles more frequent in males and lumbar hyperlordosis more prevalent in females.^35,36^ Adolescence is a period of intense physical transformation, and postural development is strongly influenced by growth peaks that occur earlier in girls (9–13 years) and later in boys (11–15 years).^37,38^

 Another limitation is the diversity in intervention duration, frequency, and supervision, which hampers the identification of optimal protocols. Moreover, although several studies such as those by Shadi et al.,^28^ Firouzjah et al.,^19^ Yang,^29^ Elpeze and Usgu,^26^ and Feng et al.^11^ reported statistically significant differences between groups, few provided measures of effect size, limiting the clinical interpretation of their results. Additionally, most studies including those by López-Ruiz and López-Miñarro,^27^ Katarzyna et al.,^32^ and Batistão et al.^31^ did not include long-term follow-up, raising concerns regarding the sustainability of the postural improvements observed. 

 With regard to assessment methods, the included studies employed a wide range of postural evaluation tools, from photographic analysis and visual inspection to spinal measurement devices. Some relied on subjective measures or functional tests, which may introduce bias and reduce comparability across studies. In several studies, even the control groups demonstrated an improvement, which could reflect test familiarity, attention bias, or insufficient separation between experimental and control activities. 

 In terms of outcomes, several authors reported improvements in specific angles or regions. Shadi et al.^28^ and Firouzjah et al.^19^ observed improvements in forward head posture, shoulder alignment, and thoracic curvature, while Yang,^29^ Elpeze and Usgu,^26^ and Feng et al.^11^ documented enhanced posture awareness and mobility. González-Gálvez et al.^14,30^ emphasized the efficacy of Pilates in reducing kyphosis and improving lumbar and pelvic alignment. Batistão et al.^31^ showed reductions in pain and improved shoulder posture. Katarzyna et al.^32^ reported significant improvements in lumbosacral inclination after Karate training, although no changes were found in thoracic or lumbar curvatures, suggesting localized adaptations. Additionally, the findings by Bansal et al.,^12^ although focused on adults, underline the importance of considering sex distribution in participation, as a higher prevalence of postural deviations has been linked to gender differences. 

 The role of schools in implementing posture-focused programs deserves attention. Interventions conducted in educational settings demonstrated improvements in knowledge, awareness, and postural behavior, contributing to the prevention of musculoskeletal disorders in adulthood.^39-41^ Since static postures during classes and screen time are associated with postural deviation,^42^ early interventions can have a preventive impact. Moreover, postural problems in youth can be reversible if corrected early,^42^ reinforcing the importance of accessible and systematic interventions in schools. 

 Future studies should adopt more rigorous designs with larger and more diverse samples, stratify participants by sex and biological maturity, and report effect sizes to support clinical relevance. Longitudinal designs are also needed to assess sustainability. Functional and psychosocial outcomes such as pain reduction, self-esteem, and academic performance should be included to provide a broader understanding of the benefits of posture-oriented programs. 

 Overall, the evidence suggests that implementing physical exercise programs in adolescents can positively impact spinal posture, particularly in improving mobility, flexibility, and reducing thoracic kyphosis and lumbar lordosis angles. Programs with a frequency of two to three sessions per week, lasting 15 to 45 minutes over a period of eight to 12 weeks, appear most effective. These findings highlight the relevance of integrating regular exercise into adolescent routines, especially in school environments, to prevent and correct postural problems during a critical stage of development. 

## Data Availability

The database that originated the article is available with the corresponding author.
